# Characterization of the Nicotianamine Exporter ENA1 in Rice

**DOI:** 10.3389/fpls.2019.00502

**Published:** 2019-04-30

**Authors:** Tomoko Nozoye, Nicolaus von Wirén, Yoshikatsu Sato, Tetsuya Higashiyama, Hiromi Nakanishi, Naoko K. Nishizawa

**Affiliations:** ^1^Center for Liberal Arts, Meiji Gakuin University, Tokyo, Japan; ^2^Graduate School of Agricultural and Life Sciences, The University of Tokyo, Tokyo, Japan; ^3^Molecular Plant Nutrition, Leibniz Institute of Plant Genetics and Crop Plant Research, Gatersleben, Germany; ^4^Institute of Transformative Bio-Molecules (WPI-ITbM), Nagoya University, Nagoya, Japan; ^5^Research Institute for Bioresources and Biotechnology, Ishikawa Prefectural University, Nonoichi, Japan

**Keywords:** iron chelator, mugineic acid, nicotianamine (NA) export, graminaceous plant, transporter

## Abstract

Under iron (Fe) deficiency, graminaceous plants produce and secrete Fe-chelating phytosiderophores of the mugineic acid (MA) family into the rhizosphere to solubilize and mediate uptake of sparingly soluble Fe in the soil. MAs and their biosynthetic intermediate, nicotianamine (NA), are also important for the translocation of divalent metals such as Fe and zinc (Zn) throughout the plant body. In this study, the physiological role of the efflux transporter EFFLUX TRANSPORTER OF NA (ENA1), which exports NA out of cells, was analyzed in rice. *Promoter-GUS* analysis showed that *ENA1* was mainly expressed in roots, and strongly upregulated under Fe-deficient conditions. In epidermal onion cells and rice roots, green fluorescent protein-tagged ENA1 localized mainly to the plasma membrane, while a part of the fluorescence was observed in vesicular structures in the cytoplasm. In the younger stage after germination, *ENA1*-overexpressing rice plants exhibited truncated roots with many root hairs compared to wild-type plants, while these phenotype were not observed in high Zn-containing medium. In *Arabidopsis*, which use a different strategy for Fe uptake from rice, *ENA1* overexpression did not show any apparent phenotypes. Oligo DNA microarray analysis in rice showed that *ENA1* knockout affects the response to stress, especially in root plastids. These results suggest that ENA1 might be recycling between the plasma membrane and cellular compartments by vesicular transport, playing an important role in the transport of NA, which is important for the physiological response to Fe deficiency.

## Introduction

Iron (Fe) is an essential element for all living organisms. In plants, Fe plays a key role in electron transfer in both photosynthetic and respiratory reactions in chloroplasts and mitochondria. Fe deficiency leads to leaf chlorosis and decreased plant yield. On the other hand, excess Fe is toxic, because it catalyzes the generation of free radicals. To acquire Fe from the rhizosphere, plants have evolved two strategies (Marschner et al., 1986). In strategy-I plants, Fe is mobilized via coumarin-type siderophores (Schmid et al., 2014; Rajniak et al., 2018; Tsai et al., 2018) and Fe(III) reduction prior to uptake in the form of ferrous Fe (Eide et al., 1996; Robinson et al., 1999; Vert et al., 2002). In strategy-II plants, Fe is mobilized via chelation through mugineic acid (MA)-type phytosiderophores (Takagi, 1976) and uptake of intact Fe(III)-phytosiderophore complexes (Römheld and Marschner, 1986). It has been believed that most plants acquire Fe by strategy-I, while only graminaceous plant species acquire Fe by strategy-II (Marschner, 1995).

In graminaceous plants, MAs are synthesized through a conserved pathway, in which three sequential enzymatic reactions convert *S*-adenosyl methionine to 2′-deoxymugineic acid (DMA) (Mori and Nishizawa, 1987; Shojima et al., 1990; Ma and Nomoto, 1993; Ma et al., 1999; Mori, 1999; Arco and Satrústegui, 2005). Expression of the genes involved in MAs biosynthesis is strongly induced by Fe deficiency. MAs are also involved in metal transport in planta (Mori et al., 1991; Kawai et al., 2001; Suzuki et al., 2006; Kakei et al., 2009). Only in the phytosiderophore biosynthesis pathway of graminaceous plants, nicotianamine (NA) acts as the direct precursor for the synthesis of DMA, whereas NA is synthesized in all plants including those that employ strategy-I for Fe acquisition (Noma and Noguchi, 1976; Rudolph et al., 1985). NA is structural analog of MAs and chelates metal cations, including Fe and zinc (Zn), manganese (Mn), and copper (Cu) (Beneš et al., 1983; Von Wirén et al., 1999). In both Strategy I and II plants, NA functions as metal chelator for intracellular metal trafficking and for long-distance metal transport between different organs (Takahashi et al., 2003; Bashir et al., 2010; Kobayashi and Nishizawa, 2012), including phloem-mediated transport of Fe from source to sink organs (Hell and Stephan, 2003; Takahashi et al., 2003; Schuler et al., 2012). In graminaceous plants, it was reported that NA is a main chelator of Zn in the phloem sap (Nishiyama et al., 2012). Intracellularly, NA was suggested to be important for vacuolar sequestration in the detoxification of excess Fe (Pich et al., 2001). In *Arabidopsis halleri*, one of the intensively studied hyperaccumulator species, NA concentrations can be extremely high (Deinlein et al., 2012), suggesting that NA plays a key role in Zn hyperaccumulation.

Fe translocation and cellular Fe transport are regulated by different transporters. MAs are secreted from the roots via TRANSPORTER OF MAs (TOM1) (Nozoye et al., 2011, 2013) to solubilize Fe in the rhizosphere. The resulting Fe(III)–MAs complexes are then taken up through the action of YELLOW STRIPE 1 (YS1) transporters (Mihashi and Mori, 1989; Curie et al., 2001; Inoue et al., 2009). Transgenic rice plants with repressed expression of *TOM2*, which is a homolog of *TOM1* and can efflux DMA, showed impaired growth because of a defect in Fe mobilization (Nozoye et al., 2015). These results corroborate that DMA solubilizes and mobilizes precipitated Fe in the apoplast. ZINC-INDUCED FACILITATOR 1 (ZIF1), a homolog of TOM1 in *Arabidopsis*, is a tonoplast-localized transporter believed to transport NA from the cytoplasm to the vacuoles (Haydon and Cobbett, 2007; Haydon et al., 2012). *ZINC-INDUCED FACILITATOR 1*-overexpressing *Arabidopsis* plants showed interveinal chlorosis and had higher Fe but lower Zn concentrations in their shoots than wild-type (WT) plants, while the opposite tendency occurred in roots (Haydon et al., 2012). These results suggested that NA is involved in the subcellular distribution and inter-organ partitioning of Fe and Zn, and that perturbing NA transport may have significant impact on Fe and Zn nutrition. However, the association between Fe, Zn, and NA, and MAs remains unclear. EFFLUX TRANSPORTER OF NA (ENA1), a rice gene belonging to the major facilitator superfamily as the TOM family and ZIF1, has been described to transport NA out of cells, when expressed in *Xenopus* oocytes (Nozoye et al., 2011). The expression of *ENA1* was strongly induced in Fe-deficient roots, suggesting that ENA1 is involved in Fe homeostasis in rice. However, its physiological function *in planta* has not been identified. In this study, different approaches were taken to investigate the physiological function of ENA1 in planta.

## Materials and Methods

### Construction of Plant Expression Vectors and Transgenic Plants

To construct the β-glucuronidase (GUS) reporter fusion genes, an 1.5-kb fragment of the 5′-upstream region of *ENA1* (*Os11g0151500*) was amplified from genomic DNA extracted from rice leaves (cv. Nipponbare) using the primer pairs 5′-AAGCTTTTGGTCCAACTCTAAGAGAT-3′ and 5′-ACTAGTCAGTGGCTTCAGAACCCTCA-3′ and ligated into the pCR4 Blunt-TOPO vector (Invitrogen). Gene fragments were then excised and subcloned into the pIG121Hm vector (Hiei et al., 1994) upstream of the GUS open-reading frame to generate the *ENA1* promoter-GUS construct. To assess the subcellular localization of *ENA1* in onion epidermal cells or rice plants, an attL/attR substrate recombination reaction between pENA1 and pH7FWG2 (Karimi et al., 2002) was used to generate the CaMV *35S promoter*–*ENA1–enhanced green fluorescent protein* (*eGFP*) cassette. To confirm subcellular localization of ENA1 in rice plants, *ENA1–GFP* or *GFP–ENA1* cassettes were cloned into the pDEST35S–sGFP binary vector (Ishimaru et al., 2005) using LR Clonase (Invitrogen) according to the manufacturer’s instructions for expression under control of the 35S promoter as described previously (Nozoye et al., 2011, 2015). *Agrobacterium tumefaciens* strain C58 carrying these constructs was used to transform rice (cv. Tsukinohikari) plants (Hiei et al., 1994).

### Histochemical Localization and Subcellular Localization of ENA1

β-Glucuronidase activity in the roots and shoots of *ENA1 promoter::GUS* transgenic plants was determined using a histochemical assay, as described previously (Inoue et al., 2003; Nozoye et al., 2011, 2015). Subcellular localization of ENA1 in onion epidermal cells or rice roots was observed as described previously (Nozoye et al., 2011, 2015). FM-64 (1 mM; Molecular Probes) was used for staining the plasma membranes by incubation of rice roots section on glass slides at room temperature. Confocal images of rice roots were acquired with a laser scanning microscope (LSM Pascal and LSM 780, Zeiss, Germany) equipped with a 10×/0.45 M27 objective. Excitation/emission wavelengths of 488 nm/490–540 nm or ∼515 nm/640 nm were used for detection of GFP or FM4-64 fluorescence, respectively.

### Characterization of *ena1* Mutants

Five *ena1 Tos17* mutant lines, namely, *NG1014*, *NG1060*, *ND1041*, *ND0824*, and *NC0379*, generated through retrotransposon insertion (Hirochika et al., 1996) were obtained from the former National Institute of Agricultural Sciences (NIAS) [https://tos.nias.affrc.go.jp/ in Japanese; present organization is Institute of Agrobiological Sciences (NARO)]. To confirm the insertion of *Tos17* into the *ENA1* gene, PCR was performed using the *Tos17* left-border specific primers (Tos17L) and *ENA1-*specific primers (pE1 for *NG1060*, pE2 for *NG1014*, pE3 for *ND8024*, pE4 for *ND1041*, and pE5 for *NC0379*) (Supplementary Table S1 and Supplementary Figure S3). When the *Tos17* fragment was inserted into *ENA1* gene, PCR fragments were expected to be amplified. To explore whether *ENA1* disruption by *tos17* was homozygous or heterozygous, PCR on genomic DNA using primers amplifying the fragment between the *tos17* insertion regions was performed. Primer sets were as follows: pE1 and pE2 for *NG1060*; pE1 and pE5 for *NG0379*; and pE1 and pE4 for *NG1014*, *ND8024*, and *ND1041*. If the *Tos17* insertion was homozygous, fragments of 1.3, 3, or 1.6 kb were not expected to be amplified by the primer sets for NG1060, NC0379, or other lines, respectively, since insertion of *tos17* into these regions would disrupt the *ENA1* sequence. When the amplified fragment was the same sizes as in WT, although they carry a *Tos17* insertion in ENA1, these plants were handled as being heterozygous.

For DNA microarray analysis, rice plants were grown hydroponically. Rice seeds (*Oryza sativa* L. cv. Nipponbare) were surface-sterilized using 2.5% sodium hypochlorite and then germinated for 1 week on Murashige and Skoog (MS) medium. After germination, the seedlings were transferred to a 20-L plastic container containing a nutrient solution of the following composition: 0.7 mM K_2_SO_4_, 0.1 mM KCl, 0.1 mM KH_2_PO_4_, 2.0 mM Ca(NO_3_)_2_, 0.5 mM MgSO_4_, 10 μM H_3_BO_3_, 0.5 μM MnSO_4_, 0.2 μM CuSO_4_, 0.5 μM ZnSO_4_, 0.05 μM Na_2_MoO_4_, and 0.1 mM Fe(III)–EDTA. The pH of the nutrient solution was adjusted daily to 5.5 with 1 M HCl. The Fe-deficiency treatment was initiated 4 weeks after germination by transferring the plants to an Fe(III)–EDTA-free nutrient solution. Experiments were performed in triplicate.

For germination analysis under high Zn conditions, rice seeds were germinated on MS medium (control) or MS medium containing 2 mM ZnSO_4_ (high Zn) according to Song et al. (2011), and cultured under a 14-h photoperiod at 320 μmol photons m^-2^ s^-1^ at 30°C. Plants were harvested at 12 days after germination, and the lengths of shoots and roots were measured. Nine plants for each line were used for each analysis. The concentrations of Fe, Zn, Mn, and phosphorus (P) were determined using inductively coupled plasma–mass spectroscopy, as described previously (Nozoye et al., 2014a).

### Semiquantitative Reverse Transcription (RT)-PCR

Total RNA was extracted from the roots of three plants per line using the RNeasy Plant Kit (Qiagen) according to the manufacturer’s instructions. Using ReverTra Ace qPCR RT Master Mix with gDNA Remover (Toyobo, Tokyo, Japan), contaminating genomic DNA was removed from total RNA and first-strand cDNA was synthesized. The forward and reverse primers used for *ENA1* were 5′-ACAAATTGGCAAGGAACTGA-3′ and 5′-CAAGATTGTGGCGTTACAAC-3′. The forward and reverse primers used for *OsActin1* were 5′-ACACCGGTGTCATGGTCGG-3′ and 5′-ACACGGAGCTCGTTGTAGAA-3′. The sizes of the amplified fragments were confirmed by agarose gel electrophoresis.

### Analysis of Rice Genes Overexpressed in *Arabidopsis thaliana*

Transgenic *Arabidopsis* plants overexpressing *ENA1*, *OsIRO2* (Ogo et al., 2006), *DMAS1*, *TOM2*, and *TOM3* generated previously by Kondou et al. (2009) were obtained from the rice full-length cDNA overexpressing *Arabidopsis* mutant database^1^ (Ichikawa et al., 2006). *Arabidopsis* seeds were surface-sterilized and germinated on 1/2 MS medium containing 100 μM Fe(III)-EDTA, 1/2 MS medium without Fe, 1/2 MS medium replacing Fe(III)-EDTA with 1 μM FeCl_2_, or 1/2 MS medium replacing Fe(III)-EDTA with 1 mM Fe(III)-citrate. Shoots were harvested 1 month after germination and used for analysis of metal concentrations as described above.

### Oligo DNA Microarray Analysis

Microarray expression analysis was performed as described previously (Nozoye et al., 2011). Total RNA was extracted from shoots and roots of *ena1* mutants (ND0824 #A, NG1060 #A, and NG1060 #D) and WT plants using the RNeasy Plant Kit (Qiagen), labeled with Cy-3 or Cy-5, and hybridized to Agilent rice 44K oligo DNA microarrays (Agilent Technologies). For each line, a mixture of three plants was used to extract RNA. For expression analysis, gene expression ratios were calculated by (signal value of *ena1* mutant sample)/(signal value of WT sample), and genes whose expression levels were increased or decreased by at least twofold were categorized as upregulated or downregulated, respectively. To assess reproducibility of the microarray analysis, genes whose expression levels were changed in all three independent lines (ND0824 #A, NG1060 #A, and NG1060 #D) were further analyzed.

To identify upregulated genes under Fe-deficient conditions, the ratio was calculated as (signal value of Fe-deficient [–Fe] sample)/(signal value of Fe-sufficient [+Fe] sample), and these ratios were used to identify Fe deficiency-induced genes, as described previously (Nozoye et al., 2011). Genes showing differential expression with a significant difference according to Student’s *t*-test (*P* < 0.05) were analyzed. To evaluate the function of *ENA1 in planta*, we performed gene ontology (GO) analysis using agriGO (Du et al., 2010; Tian et al., 2017). agriGO is designed to identify enriched GO terms in a list of microarray probe sets^2^. Three categories were included in the analysis, namely biological process, molecular function, and cellular component.

## Results

### Promoter-GUS Analysis of ENA1

**FIGURE 1:**
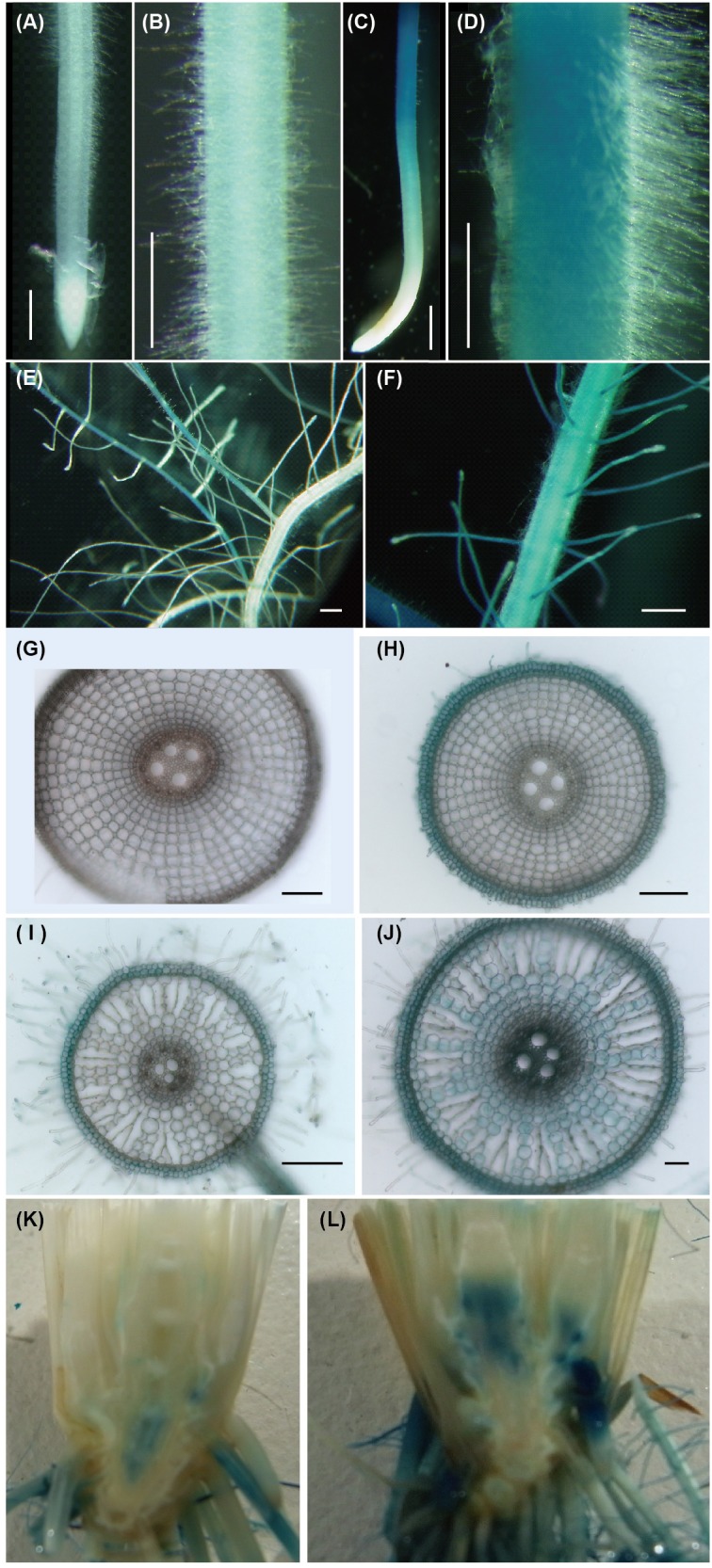
Promoter activity of ENA1 in rice during vegetative growth as indicated by GUS staining. **(A,B,E,G,I)** Iron (Fe)-sufficient root. **(C,D,F,H,I)** Fe-deficient root. **(G,H)** Cross-sections of the elongation zone. **(I,J)** Cross-sections of the root near the basal part. **(K,L)** Tissue distribution of *ENA1* promoter activity in root–shoot junctions under Fe-sufficient **(K)** or Fe-deficient **(L)** conditions. The basal part of the shoot was cut vertically and the interior tissue is shown. Scale bars: 1 mm **(A–F)**; 200 μm **(G–J)**.

In Fe-sufficient plants, *ENA1* promoter activity was low in primary and branched lateral roots (Figure 1A,B,E), where *ENA1* expression was localized to the epidermis and cortex (Figure 1G,I). Under Fe-deficient conditions, *ENA1* promoter activity was strongly enhanced in both, primary and lateral roots, except in root tips (Figure 1C,D,F,H,J). Root cross-sections further revealed that *ENA1* promoter activity was confined to the epidermis near the root tips, while it was detected across all tissues in basal root zones (Figure 1H,J). In shoots, *ENA1* expression was also induced by Fe deficiency but only observed near the root-to-shoot junction, where translocation spots exist between xylem and phloem (Figure 1K,L). In leaves, *ENA1* expression was not observed (data not shown).

### Intracellular Localization of ENA1

**FIGURE 2:**
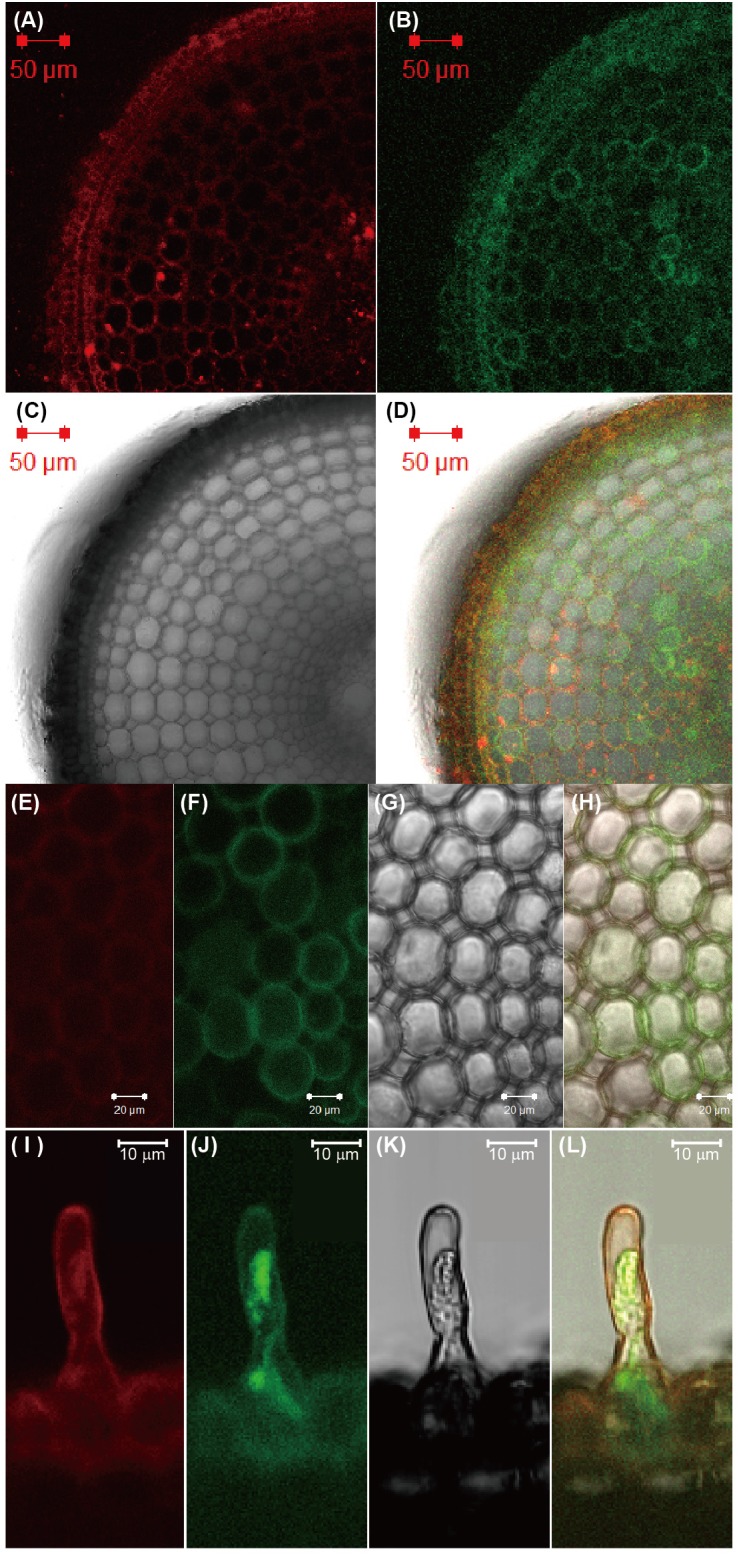
Subcellular localization of ENA1-GFP in rice roots. **(A,E,I)** Fluorescence image of FM65-stained rice roots. **(B,F,J)** Fluorescence image of GFP. **(C,G,K)** Differential interference contrast image. **(D,H,L)** Overlay.

To investigate the subcellular localization of ENA1, *ENA1–GFP*, or *GFP–ENA1* fusion constructs were transiently expressed in onion epidermal cells (Supplementary Figure S1). Both ENA1–GFP and GFP–ENA1 fusion proteins were observed in the plasma membrane and in the cytoplasm. In the cytoplasm, some of the GFP–ENA1 and ENA1–GFP fusion proteins were localized to vesicular structures. This localization was more common with the ENA1–GFP fusion protein than the GFP–ENA1 fusion protein. To confirm the cellular localization in rice plants, *ENA1–GFP* or *GFP–ENA1* fusion genes were stably expressed in rice plants, in which expression was observed in roots. There ENA1–GFP-dependent fluorescence was mainly localized to the plasma membrane (Figure 2 and Supplementary Figures S2A–F). A part of ENA1–GFP fusion proteins was observed in intracellular compartments. The fluorescence intensity from the GFP–ENA1 fusion protein was much weaker than that of the ENA1–GFP fusion, while the mRNA levels of *GFP–ENA1* and *ENA1–GFP* fusion genes were similar (Supplementary Figure S2G). The expression pattern was not affected by the Fe concentration of the medium.

### Analysis of the tos17 Insertion Line in ENA1 Loci

**FIGURE 3:**
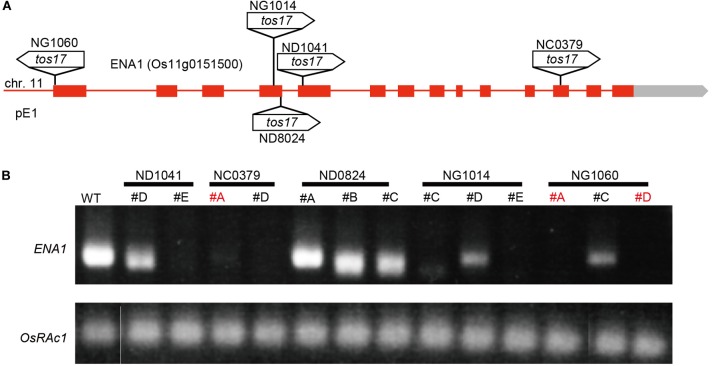
*Tos17* insertion positions and *ENA1* expression in the mutants. **(A)** Schematic representation of *ENA1* and insertion positions of the *tos17* fragments. **(B)** RT-PCR was performed to verify the expression of *ENA1* in *ena1 tos17* insertion lines. The *ena1* knockout mutant lines NC0379#A, and NG1060#A and #D, were further analyzed.

Five *tos17* insertion mutants with retrotransposon fragments inserted in *ENA1* (NG1014, NG1060, ND1041, ND0824, and NC0379) were identified (Figure 3A and Supplementary Figure S3A). Nested PCR was performed to confirm the insertion of *tos17* in *ENA1*, which showed that three lines of NG1014 (#C, #D, #E), three of NG1060 (#A, #C, #D), two of ND1041 (#D, #E), two of ND0824 (#B, #C), and two of NC0379 (#A, #D) carried a *tos17* insertion in the *ENA1* gene (Supplementary Figure S3B). Next, to explore whether the transgenic plants harboring the *tos17* retrotransposon in *ENA1* were in a homozygous or heterozygous state, genomic PCR amplifying the *tos17* insertion region was performed (Supplementary Figure S3C). NG1014 (#D), NG1060 (#A, #C, #D), ND1041 (#D, #E), and NC0379 (#A) were heterozygous, while NG1014 (#C, #E), ND0824 (#B, #C), and NC0379 (#D) were homozygous. RT-PCR showed that *ENA1* was knocked-out in NG1014 (#C, #E), NG1060 (#A, #D), ND1041 (#E), and NC0379 (#A, #D) (Figure 3B and Supplementary Figure S3B). *ENA1* fragments in ND0824 (#B, #C) were not absent, although the *tos17* insertion in *ENA1* was homozygous, and their sizes were smaller than in the WT. In ND0824, *Tos17* was inserted at the junction of the intron and exon of *ENA1*, which could influence the size of *ENA1*.

To analyze the function of ENA1 in planta, plants were cultivated hydroponically under Fe-sufficient or Fe-deficient conditions, and plant characteristics such as plant height, root length, chlorophyll content, and metal concentrations were analyzed (Supplementary Figure S4). However, there were no apparent differences among *ena1* knockout mutants, *ENA1–GFP-* or *GFP–ENA1-*overexpression lines, or WT plants.

**FIGURE 4:**
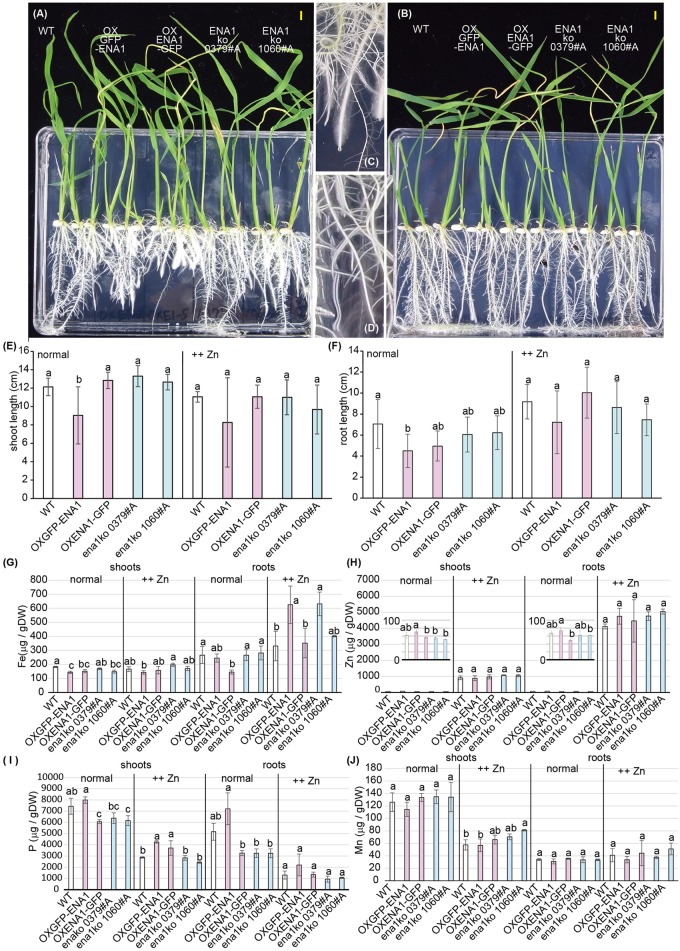
Plant phenotype of *ena1* knockout mutants (0379#A and 1060#A), *GFP-ENA1*- or *ENA1-GFP*-overexpressing rice plants, and WT. Rice plants grown on MS medium (normal) or MS medium with high zinc (Zn) concentrations (++Zn). **(A–D)** Plant appearance at 12 days after germination under normal **(A,C,D)** or high (Zn **(B)** conditions. Scale bars represent 0.5 cm. Roots pictures of *ENA1-GFP* overexpressing **(C)** or wild-type (WT) plants **(D)** under normal conditions are enlarged. **(E,F)** Shoot length **(E)** and root length **(F)** were measured 12 days after germination. Error bars represent the standard error (*n* = 9). **(G–J)** Fe, Zn, manganese (Mn), and phosphorus (P) concentrations in the shoots and roots of *ena1* knockout mutants, *GFP-ENA1*- or *ENA1-GFP*-overexpressed rice plants, and WT. Values represent means of three replicates. Error bars represent standard deviations. DW, dry weight. Different letters indicate significant differences according to the Tukey–Kramer HSD test (*n* = 9, *P* < 0.05).)

Since ENA1 has the ability to export NA (Nozoye et al., 2011), it was speculated that the function of ENA1 *in planta* might be similar to that of ZIF1, a putative NA exporter in *Arabidopsis*, and involved in Fe and Zn homeostasis (Haydon and Cobbett, 2007; Haydon et al., 2012). Interveinal chlorosis of *ZIF1*-overexpressing Arabidopsis was recovered by addition of Fe, while this phenotype became more severe under high Zn conditions (Haydon et al., 2012). Therefore, the phenotypes of *ena1* knockout mutants in rice, NG1060 #A and NC0379 #A, the *ENA1–GFP-* or *GFP–ENA1*-overexpressing lines, and WT were compared in MS medium with adequate nutrient supply or excess Zn supply (Figure 4). Under adequate nutrient supply, roots of *ENA1–GFP*- and *GFP–ENA1*-overexpressing rice plants were shorter than those of WT and *ena1* knockout mutants (Figure 4A,F). The*ENA1–GFP-* and *GFP–ENA1*-overexpressing plants had more root hairs compared to WT and *ena1* knockout mutants (Figure 4A,C,D). Under high Zn conditions, the short-root phenotype and enhanced root hair formation of *ENA1–GFP*- and *GFP–ENA1*-overexpressing rice plants were no longer observed (Figure 4B). These phenotypes were not observed when the plants were grown hydroponically (Supplementary Figure S4E). Under adequate nutrient supply, Fe concentrations in shoots were lower in *ENA1*-overexpressing rice plants and *ena1* knockout mutant 1060#A compared to WT (Figure 4G). Under adequate nutrient supply, root Fe concentrations in *ENA1–GFP*-overexpressing rice plants were slightly lower than in WT and *ena1* knockout plants. Under high Zn conditions, Fe concentrations in the shoots were not significantly different among *ENA1*-overexpressing rice plants, *ena1* knockout mutants, and WT plants. Under high Zn conditions, root Fe concentrations in *GFP–ENA1*-overexpressing rice plants and *ena1* knockout mutants 1060#A were slightly higher compared with WT plants. Zn concentrations did not significantly differ among *ENA1*-overexpressed rice plants, *ena1* knockout mutants, and WT under both adequate nutrient supply and high Zn condition (Figure 4H). Under adequate nutrient supply, P concentrations in shoots were slightly lower in *ENA1–GFP*-overexpressing rice plants and the *ena1* knockout line 1060#A compared to WT (Figure 4I). Under high Zn conditions, shoot P concentrations were slightly higher in both *ENA1–GFP* and *ENA1–GFP* overexpressing rice plants compared to WT and *ena1* knockout mutants. In roots, there was no difference in P concentrations among these lines. There were no differences in Mn concentrations following modification of *ENA1* expression (Figure 4J).

### Analysis of ENA1-Overexpressing Arabidopsis

**FIGURE 5:**
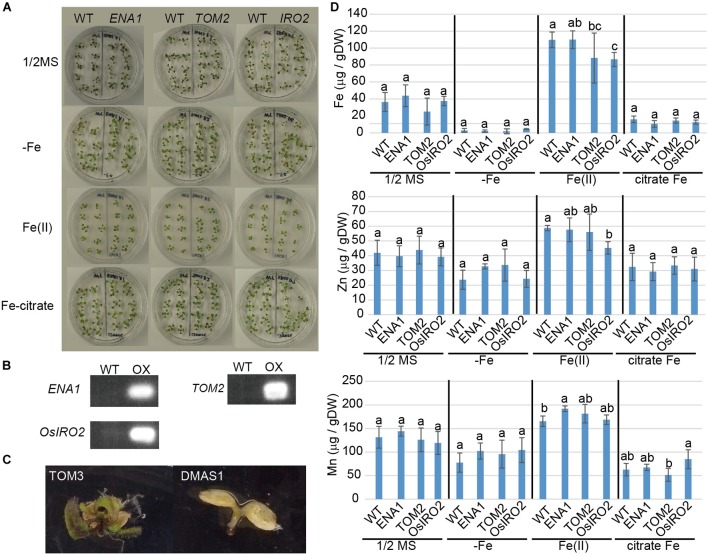
Phenotype of transgenic *Arabidopsis* plants overexpressing *ENA1*, *TOM2*, *TOM3*, *OsIRO2*, or *OsDMAS1*. **(A)** Plant appearance at 1 month after germination. *ENA1-*, *TOM2-*, or *OsIRO2*-overexpressing *Arabidopsis* plants were grown next to WT plants in the same medium. **(B)** RT-PCR analysis of the expression of *ENA1*, *TOM2*, and *OsIRO2* in *Arabidopsis* shoots. **(C)** Plant appearance of *Arabidopsis* plants overexpressing *TOM3* or *OsDMAS1* at 1 month after germination. **(D)** Fe, Zn, and Mn concentrations in the shoots of transgenic *Arabidopsis* overexpressing *ENA1*, *TOM2*, or *OsIRO2*. Values are the means of three to six replicates. Error bars represent standard deviation. DW, dry weight. Letters indicate significant differences according to Tukey–Kramer HSD test (*n* = 3–6, *P* < 0.05).

*ZINC-INDUCED FACILITATOR 1*-overexpressing *Arabidopsis* exhibited a drastic phenotypes with interveinal chlorosis and higher shoot Fe (Haydon and Cobbett, 2007; Haydon et al., 2012). To examine whether this phenotype could be reproduced by overexpression of *ENA1*, transgenic *Arabidopsis* plants overexpressing *ENA1* were analyzed (Figure 5). Transgenic *Arabidopsis* plants overexpressing rice Fe homeostasis-related genes including *OsIRO2*, *TOM1*, *TOM2*, *TOM3*, and *OsDMAS1* were also analyzed. The plant appearance of *ENA1-*, *OsIRO2-*, and *TOM2*-overexpressing *Arabidopsis* was not different from WT, irrespective of Fe treatment (Figure 5A). One month after germination, plants were harvested and the metal concentrations in shoots were analyzed (Figure 5C). No significant differences in shoot Fe, Zn, or Mn concentrations were observed between WT plants and transgenic plants overexpressing *ENA1*, *OsIRO2*, and *TOM2*, independent of external Fe treatment. Mn concentrations in *ENA1-*overexpressing *Arabidopsis* shoots were higher than in WT plants when grown with an Fe(III) source. Fe and Zn concentrations in *OsIRO2-*overexpressing *Arabidopsis* shoots were lower than in WT plants when grown with an Fe(III) source. *Arabidopsis* plants overexpressing *DMAS1* and *TOM3* exhibited impaired germination and were unable to survive (Figure 5C). *TOM1*-overexpressing *Arabidopsis* seeds did not germinate, although the seed age was similar to that of WT and other transgenic *Arabidopsis* seeds (data not shown).

### Oligo DNA Microarray Analysis of *ena1* Knockout Mutant Lines

**FIGURE 6:**
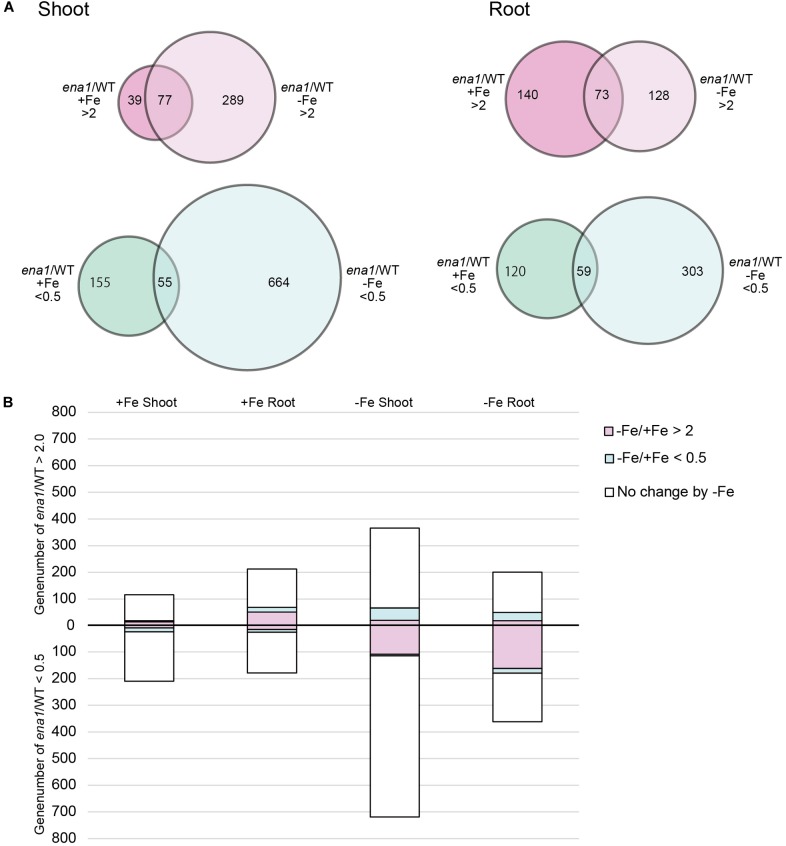
Gene expression analysis in *ena1* knockout mutants and WT plants. **(A)** Venn diagrams represent genes differentially expressed in roots of *ena1* knockout mutants compared to WT plants under Fe-sufficient or Fe-deficient conditions. Of a total of 29,864 genes, 2,102 (746 upregulated and 1,356 downregulated) were differentially expressed by at least twofold between *ena1* knockout mutants compared to WT plants under both Fe-sufficient and Fe-deficient conditions compared with the gene list in Supplementary Table S2. **(B)** Graphs representing the numbers of Fe deficiency-responsive genes among the 2,102 genes, whose expression levels were upregulated or downregulated in *ena1* knockout mutants compared to WT shoots and roots under Fe-sufficient or Fe-deficient conditions. Pink or blue colors indicate the ratios of genes that showed upregulation (–Fe/+Fe > 2) or downregulation (–Fe/+Fe < 0.5), respectively, by at least twofold under Fe-deficient conditions in the WT.

To analyze the molecular function of *ENA1 in planta*, genome-wide changes in shoot and root gene expression were compared in *ena1* knockout and WT plants using oligo DNA microarray analysis. To exclude the effect of *Tos17* insertions in the genome other than in the *ENA1* locus, only those genes were analyzed, whose expression level was at least twofold upregulated or downregulated in all plants of two *ena1* knockout mutants (NC0379#A, NG1060#A, and NG1060#D). Among the 29,864 unique genes on the oligo DNA microarray, 116 and 213 genes were upregulated in shoots and roots, respectively, of the *ena1* knockout mutants under Fe-sufficient conditions (Figure 6A). Under Fe deficiency, 366 and 201 genes were upregulated in *ena1* shoots and roots, respectively. In contrast, 210 and 179 genes were downregulated in shoots and roots of the *ena1* knockout mutants under Fe-sufficient conditions, while 719 and 362 were downregulated in *ena1* shoots and roots, respectively, under Fe-deficient conditions. The upregulated and downregulated genes in shoots and roots under both Fe-sufficient and Fe-deficient conditions of the *ena1* knockout mutants compared to WT are listed in Tables 1, 2. In the list of upregulated genes, almost all genes are unknown and have not yet been characterized (Table 1). On top of the downregulated genes in the *ena1* knockout mutants was a gene homologous to PV72, a seed-specific vacuolar sorting receptor (Watanabe et al., 2004), whose expression was downregulated in roots and upregulated in shoots of the WT by Fe deficiency (Table 2).

**Table 1:** Upregulated genes in the *ena1* knockout mutants.

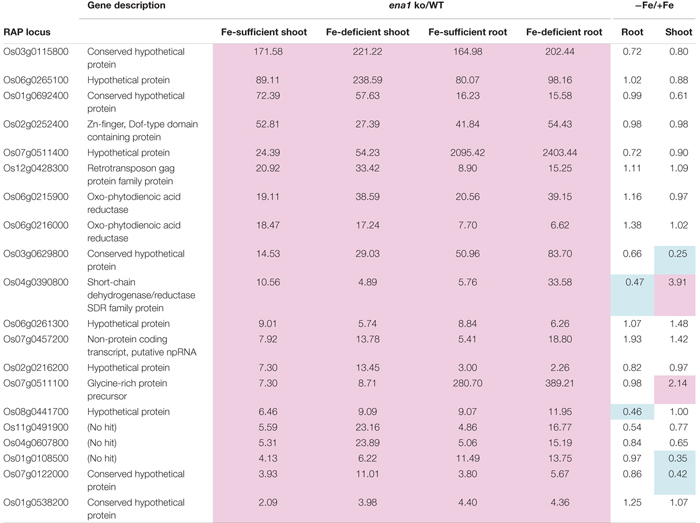

*Listed are the genes whose expression levels were upregulated at least twofold in the *ena1* knockout (ko) mutants compared to wild type (WT). Shown are the averages of the expression ratios calculated by (signal value of the *enal* ko mutants line)/(signal value of the corresponding WT) in the three *ena1* ko lines (NC0379#A, NG1060#A, and NG1060#D). The ratios calculated by (signal value in WT under Fe-deficient condition)/(signal value in WT under Fe-sufficient condition) are also represented. Upregulated or downregulated genes are shaded in pink or blue, respectively.*

**Table 2:** Downregulated genes in the *ena1* knockout mutants.

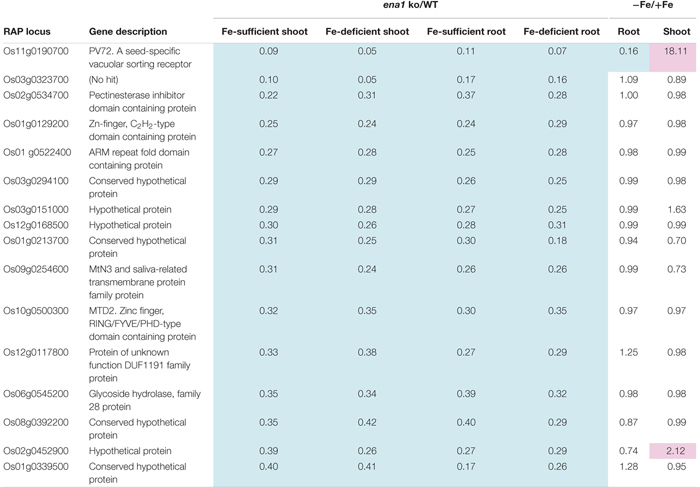

*Listed are the genes whose expression levels were downregulated by at least twofold in the *ena1* knockout (ko) mutants compared to WT. Shown are the averages of the expression ratios calculated by (signal value of the *enal* ko mutants line)/(signal value of the corresponding WT) in the three *ena1* ko lines (NC0379#A, NG1060#A, and NG1060#D). The ratios calculated by (signal value in WT under Fe-deficient condition)/(signal value in WT under Fe-sufficient condition) are also presented. Upregulated or downregulated genes are shaded in pink or blue, respectively.*

Under Fe deficiency, we observed significant changes in gene expression, reflecting how plants cope with the stress and maintain Fe homeostasis. Therefore, it was analyzed whether the expression of Fe deficiency-regulated genes was altered in *ena1* knockout mutants (Figure 6B). Among the genes whose expression was different from the WT in *ena1* knockout mutants, 13.5% and 16.8% of genes in shoots under Fe-sufficient and Fe-deficient conditions, respectively, were genes modulated by Fe deficiency (Figure 6B). In roots, the corresponding numbers were 24.2% and 40.9%. Among the Fe deficiency-modulated genes, whose expression was up-regulated in *ena1* knockout mutants compared with the WT under Fe-sufficient conditions, the ratio of the upregulated genes by Fe deficiency was higher than that of the down-regulated genes, especially in the roots. Under Fe-deficient condition, this trend was opposite. The ratio of downregulated genes by Fe deficiency was higher than that of upregulated genes among the upregulated genes in the *ena1* knockout mutants compared with WT, while the ratio of upregulated genes by Fe deficiency was higher than that of down-regulated genes by Fe deficiency among the down-regulated genes in the *ena1* knockout mutants compared with WT. Expression changes of Fe homeostasis-related genes in the *ena1* knockout mutants compared with WT were analyzed (Table 3). The expression of *OsNAS1*, *OsNAS2*, *OsDMAS1*, *TOM1*, and *OsYSL15* tended to be lower in Fe-deficient shoots of *ena1* knockout mutants than of WT plants.

**Table 3:** Expression changes of Fe homeostasis-related genes in the *ena1* knockout (ko) mutants.

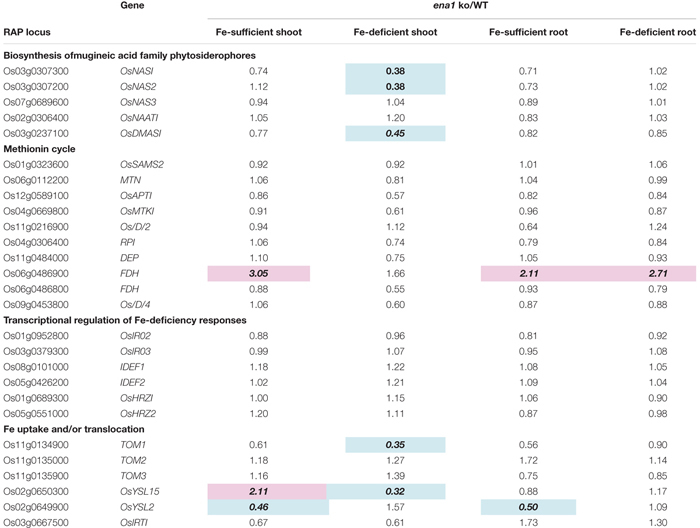

*Listed are Fe deficiency-inducible genes involved in Fe uptake and utilization and transcriptional regulators of Fe-deficiency responses. Shown are the averages of the expression ratios calculated by (signal value of the *enal* ko mutants line)/(signal value of the corresponding WT) in the three *ena1* ko lines (NC0379#A, NG1060#A, and NG1060#D). Upregulated or downregulated genes are shaded in pink or blue, respectively. Ratios upregulated or downregulated by at least twofold in all three lines are indicated in bold, while if at least one replicate was not upregulated or downregulated by at least twofold, values are indicated in italics*

To gain further insights into ENA1-dependent processes, an agriGO analysis was carried out to identify enriched categories among the genes, whose expression level was altered in the *ena1* knockout mutants (Supplementary Figures S5–8). In Fe-deficient roots of *ena1* plants, the most significantly enriched GO term for biological processes was “photosynthesis, light harvesting” (GO:0009765) (Supplementary Figure S5A). GO enrichment analysis also revealed enrichment of genes involved in the biological processes “oxylipin metabolism,” “jasmonic acid biosynthesis,” and “systemic acquired resistance.” The molecular function “tetrapyrrole binding” (GO:0046906) (Supplementary Figure S5B) and the cellular compartments “membrane-bound vesicle” (GO:0031988) and “cytoplasmic membrane-bound vesicle” (GO:0016023) (Supplementary Figure S5C) were also strongly enriched among the genes downregulated in Fe-deficient roots of the *ena1* knockout. In Fe-deficient shoots of *ena1* knockout mutants, “DNA replication” (GO:0006260) and “methylammonium transmembrane transporter activity” (GO:0015200) were the only significantly enriched terms in biological process and molecular function, respectively (Supplementary Figures S6A,B). Interestingly, “membrane-bound vesicle” (GO:0031988) and “cytoplasmic membrane-bound vesicle” (GO:0016023) were also the most significantly enriched GO terms for cellular compartments among genes downregulated in Fe-deficient shoots of the *ena1* knockout mutants (Supplementary Figure S6C). The molecular function “Zn ion binding” (GO:0008270) was enriched in the Fe-sufficient shoots of the *ena1* knockout mutants (Supplementary Figure S8A), whereas the cellular compartments “membrane-bound vesicle” (GO:0031988) and “cytoplasmic membrane-bound vesicle” (GO:0016023) were again the most significantly enriched GO terms in both Fe-sufficient roots (Supplementary Figure S7) and shoots (Supplementary Figure S8B). These GO terms were not enriched among the genes upregulated in the *ena1* knockout mutants compared to WT.

## Discussion

Since the discovery of MAs, we have explored strategies to acquire insoluble Fe from the soil, and meanwhile the molecular machinery involved in Fe acquisition in graminaceous plants has been largely identified (Kobayashi et al., 2018). MAs and NA are involved in the translocation and intracellular transport of metals, including Fe (Kakei et al., 2009; Nishiyama et al., 2012), however, the intracellular transport of Fe by MAs and NA remains largely unexplored. It has been suggested that the transporters for MAs and NA are involved in the regulation of metal flows *in planta*. In this study, we characterized ENA1, which can export NA out of the cell (Nozoye et al., 2011).

### ENA1 May Be Involved in the Transport of NA From the Intracellular Compartment Outside Cells in Roots

*Promoter-GUS* analysis showed that under Fe-sufficient conditions *ENA1* is mainly expressed in lateral roots (Figure 1). In root cross-sections, *ENA1* expression was observed in the root epidermis. Under Fe-deficient conditions, *ENA1* expression was strongly induced in roots, except near the root tip. Near the root tips, *ENA1* expression was observed only in the epidermis, while *ENA1* expression was observed in whole roots including the epidermis, cortex, and vascular bundle near the basal area of the roots. These results are consistent with previous studies, in which laser micro-dissection analysis showed that *ENA1* was mainly expressed in roots and expression in the cortex was induced by Fe deficiency (Ogo et al., 2014). The expression patterns of *ENA1* in roots were similar to those of the DMA exporters *TOM1* and *TOM2*, and of the Fe importers *OsYSL15* and *OsIRT1* (Ishimaru et al., 2006; Inoue et al., 2009; Nozoye et al., 2011, 2015). In the leaf and stem, *ENA1* expression was not observed under Fe-sufficient or Fe-deficient conditions. Interestingly, *ENA1* expression was observed in the discrimination center (DC) at the basal part of the shoots, where Fe accumulates after absorption and translocation from xylem to phloem inbarley (Tsukamoto et al., 2009). *ENA1* expression in the DC was strongly induced by Fe deficiency. *TOM2*, a DMA exporter and homolog of *TOM1*, is strongly expressed in the DC under both Fe-sufficient and Fe-deficient conditions (Nozoye et al., 2015). *TOM2* repression in rice plants impaired plant growth, suggesting that *TOM2* is involved in DMA transport, which is important for Fe mobilization in the plant body. These results suggested that Fe mobilization in the DC is important for Fe homeostasis. ENA1 may be involved in Fe mobilization in the DC by NA efflux.

**FIGURE 7:**
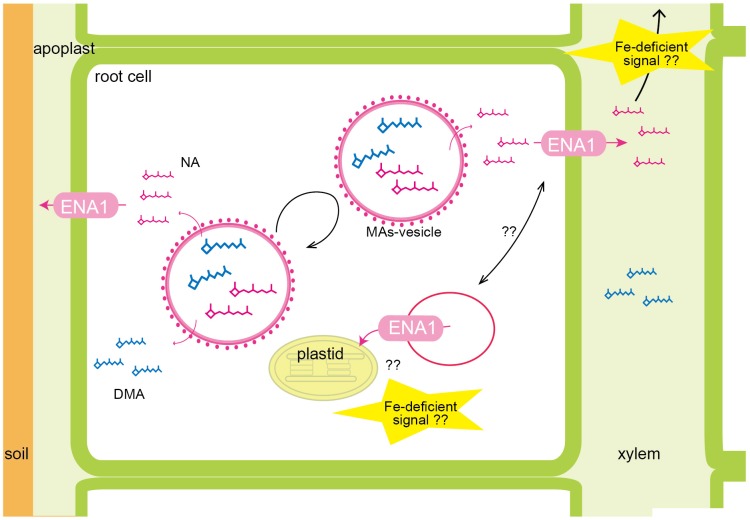
Schematic of the putative roles of ENA1 in rice. NA and DMA are predicted to be produced in intracellular vesicles, recognized as MAs-synthesizing vesicle. Since MAs-synthesizing vesicles are surrounded by rough endoplasmic reticulum, NA and DMA are exported into the cytoplasm, and then transported out of the cell via ENA1. ENA1 is mainly localized to the plasma membrane, where it transports NA to the apoplast. Regarding an intracellular function, ENA1 might export NA from vesicles for subsequent Fe loading of plastids. ENA1 transport functions are upregulated under Fe deficiency.

The subcellular localization of ENA1 was examined in onion epidermal cells (Supplementary Figure S1) and rice root cells (Figure 2 and Supplementary Figure S2). GFP–ENA1 and ENA1–GFP fusion proteins were localized in the plasma membrane and in the cytoplasm of onion epidermal cells. There, the GFP–ENA1 and ENA1–GFP fusion protein partly localized to vesicular structures. In rice roots, the ENA1–GFP fusion protein localized mainly to the plasma membrane, and partially to intracellular vesicular structures. MA biosynthesis through NA has been proposed to occur in intracellular vesicles (Nishizawa and Mori, 1987; Negishi et al., 2002; Nozoye et al., 2004, 2014a,b). MA secretion in barley and rice follows a diurnal rhythm and occurs 2–3 h after sunrise while its production is constant (Takagi et al., 1984; Nozoye et al., 2014a). These observations suggested that MAs are stored in cells until secretion. Vesicles surrounded by ribosomes have been shown to accumulate near the plasma membrane just before sunrise in Fe-deficient barley and rice roots (Nishizawa and Mori, 1987; Negishi et al., 2002; Nozoye et al., 2014a). These vesicles are not observed in Fe-sufficient roots. Therefore, these vesicles were considered to contain MAs, and to store these until secretion. It has been proposed that all enzymes involved in MAs biosynthesis localize to these vesicles. Indeed, a fusion protein of GFP with the rice NA synthase 2 (OsNAS2), one of the MAs biosynthetic enzymes, was observed as dot-like structures in rice root cells, suggesting that at least NA is synthesized in MAs-containing vesicles (Nozoye et al., 2014a). In this study, ENA1–GFP mainly localized to the plasma membrane. Since MAs-synthesizing vesicles were supposed to be surrounded by rER, it was thought that MAs-synthesizing vesicles are not fused to the plasma membrane. It was speculated that NA and/or DMA produced in MAs-synthesizing vesicles were secreted to the cytoplasm and then transported out of cells. ENA1 might be involved in the efflux of NA from the cytoplasm to the apoplast (Figure 7).

In the cytoplasm, ENA1–GFP was observed in part also vesicular structures, which differed from the dot-like structures observed for NAS2-GFP, since the number and form of vesicles differed. ENA1 fused to GFP at the N-terminus (ENA1–GFP) also localized to the plasma membrane and cellular vesicular structures. However, in onion epidermal cells the number of vesicular structures was higher for ENA1–GFP than GFP–ENA1. The fluorescence of GFP–ENA1 in rice roots was too weak to be detected, suggesting that GFP might interfere with the intracellular localization of ENA1 in rice cells. Both *ENA1–GFP-* and *GFP–ENA1*-overexpressing plants showed truncated roots with large number of root hairs, when plants were grown in MS medium with adequate Fe supply (Figure 4), suggesting that ENA1–GFP and GFP–ENA1 might be functional in rice plants. However, the changes in metal concentrations differed among *ENA1–GFP* and *GFP–ENA1*-overexpressing plants. This may be a consequence of protein mistargeting due to protein overload. To evaluate the phenotypes of *ENA1–GFP* and *GFP–ENA1*-overexpressing plants, rice plants overexpressing *ENA1* without a *GFP* tag would be required. Further analyses are also necessary to confirm the intracellular localization of ENA1.

### The Mechanism of NA Transport Differs Between Rice and Arabidopsis

To examine the physiological role of ENA1 *in planta*, characteristics of *ena1* knockout mutants carrying *tos17* insertions (Supplementary Figure S3) and *ENA1–GFP-* or *GFP–ENA1*-overexpressing rice plants was analyzed (Figure 3). However, in hydroponics there were no apparent differences (Supplementary Figure S4). In rice, NA is an intermediate of MAs biosynthesis and DMA is the final product. DMA and NA are involved in metal translocation in the plant body (Suzuki et al., 2006; Kakei et al., 2009; Nishiyama et al., 2012; Nozoye et al., 2015). On the other hand, *Arabidopsis* produces NA but not DMA. In rice, transporters of DMA, such as members of the TOM family, may compensate or even take over transport functions that are mediated by ENA1 in Arabidopsis. Such a view is supported by the fact that YS1-type transporters also transport both, DMA and NA (Schaaf et al., 2004).

Under Fe-adequate conditions, root growth of *ENA1–GFP-* or *GFP–ENA1-*overexpressing rice plants was significantly impaired, but this impairment was not observed under high Zn conditions (Figure 4). The roots of *ENA1–GFP-* or *GFP–ENA1-*overexpressing rice plants were shorter and had more root hairs than the WT. Fe concentrations were slightly lower in shoots of *ENA1–GFP* or *GFP–ENA1* overexpressing rice plants. However, these phenotypes were rather moderate compared to those of *ZIF1*-overexpressing *Arabidopsis* or *zif1* mutants (Haydon and Cobbett, 2007; Haydon et al., 2012). Since the root phenotype is similar to the response under P starvation (Peret et al., 2014), we determined P concentrations (Figure 4). However, differences in root P concentrations were not correlated with changes in *ENA1* expression. In shoots, the P concentration in *ENA1–GFP*-overexpressing rice plants was slightly lower than in WT plants under adequate nutrient supply, but slightly higher than in WT plants under elevated Zn supply, although these differences were not large. NA transport by ENA1 may be affected in part by both, Fe and P nutrition *in planta*.

When *ENA1* was overexpressed in *Arabidopsis*, plants grew well and metal concentrations in the leaves were not significantly different from WT, unlike under overexpression of ZIF1. A difference was only observed in the Mn concentration of shoots. Based on amino acid homology, ZIF1 is most closely related to TOM1, which may be the main transporter responsible for DMA secretion from roots into rhizosphere (Nozoye et al., 2011). However, it has been shown that TOM1 can efflux DMA, but not NA. In rice, *TOM2* and *TOM3* are homologs of *TOM1*. It has been shown that TOM2 is also a DMA exporter that plays a role in metal translocation in the plant body (Nozoye et al., 2015). Since both NA and DMA are involved in long-distance metal translocation, TOM2 might complement ENA1 functions in rice. In this study, similar to the case with ENA1, *TOM2*-overexpressing *Arabidopsis* did not show any obvious phenotypes (as opposed to *ZIF1*-overexpressing *Arabidopsis*), while *TOM1*-, *TOM3*-, and *OsDMAS1*-overexpressing *Arabidopsis* could not germinate. *Arabidopsis* NAS (AtNAS1-4) did not localize to vesicular structures in the cytoplasm like OsNAS2-GFP did in rice (Nozoye et al., 2014b). These results suggest that the cellular compartments producing NA may differ between rice and *Arabidopsis*, and these cellular compartments may be relevant for the function of NA.

### Oligo DNA Microarray Analysis Suggests That Intracellular Transport of NA Through ENA1 May Be Involved in Maintaining Fe Homeostasis, and ENA1 Knockouts Influence Plastid Function in Roots

To examine the molecular function of *ENA1*, oligo DNA microarray analysis was performed. In *ena1* knockout mutants, the number of genes whose expression level was downregulated was larger than that of upregulated genes relative to the WT. Previously, we identified genes whose expression was upregulated under Fe deficiency, including transcription factors, MAs biosynthetic enzymes, and Fe transporters (Nozoye et al., 2011, 2014a). Under Fe-sufficient conditions, genes whose expression was induced under Fe deficiency tended to be upregulated in *ena1* knockout mutants compared to WT (Figure 6B). By contrast, under Fe-deficient conditions, genes whose expression level was induced by Fe deficiency tended to be downregulated in *ena1* knockout mutants (Figure 6B). Indeed, the expression levels of the genes involved in MAs biosynthesis and transport were lower in the Fe-deficient shoots (Table 3). These results suggest that *ena1* mutants respond to Fe deficiency even under Fe-sufficient conditions; however, *ena1* mutants could not respond to Fe deficiency under Fe-deficient conditions. agriGO analysis showed that genes whose expression levels were downregulated in *ena1* mutant roots under Fe-deficient conditions are involved in photosynthesis, jasmonic acid biosynthetic processes, and innate immunity. Further molecular functions of these genes are related to chlorophyll binding. In addition, cellular components were involved in cytoplasmic membrane-bound vesicles. Fe is important for photosynthesis in chloroplasts since it plays a role as enzyme cofactor in chlorophyll biosynthesis. In *Arabidopsis*, it has been shown that chlorophyll biosynthesis in leaves is downregulated and metabolite changes under Fe-deficient conditions allow plants to prevent photo-oxidative damage (Rodriguez-Celma et al., 2013). In general, plastids synthesize chlorophylls, carotenoids, and fatty acids, and are involved in aromatic amino acid biosynthesis, and reduce several inorganic nutrients (Buchanan et al., 2015). As some of these Fe-dependent functions are also relevant for root plastids, Fe must be imported also into root plastids. NA transport through ENA1 may take over such a function to adjust metabolism and prevent the stress response under Fe deficiency, such as the production of reactive oxygen species (Figure 7). As mentioned above, OsNAS2-GFP fusion proteins localized as dot-like structures in rice root cells (Nozoye et al., 2014a). In this study, the expression levels of *OsNAS1*, *OsNAS2*, and *OsDMAS1* were lower in the Fe-deficient shoots of *ena1* knockouts than in the WT (Table 3), suggesting that ENA1 expression is involved in DMA and NA biosynthesis, which may take place in intracellular vesicles. Other than WT plants, *ena1* mutants could not induce Fe deficiency-inducible genes under Fe deficiency. This result may indicate that ENA1 is involved in Fe trafficking into cellular compartments such as plastids. Further studies on the intracellular trafficking of Fe by Fe transporters including ENA1 will be important to characterize the mechanisms maintaining Fe homeostasis *in planta*.

## Author Contributions

TN, HN, and NN designed the research. TN carried out the experiments with assistance from NvW, YS, and TH, and analyzed the data. TN wrote the manuscript with contributions and discussion from all of the co-authors.

## Conflict of Interest Statement

The authors declare that the research was conducted in the absence of any commercial or financial relationships that could be construed as a potential conflict of interest.

## Footnotes

1 http://ricefox.psc.riken.jp/index.php?contents=top&subcontents=research&research=foxh

2 http://bioinfo.cau.edu.cn/agriGO/index.php

## Supplementary Material

Figure S1 Subcellular localization of ENA1 in onion epidermal cells. **(A)** GFP-ENA1. **(B)** ENA1-GFP.

Figure S2 Subcellular localization of ENA1-GFP in rice roots. **(A,D)** Differential interference contrast image. **(B,E)** Fluorescence image. **(C,F)** Overlay. **(G)** GFP expression was examined by RT-PCR in *ENA1-GFP*- or *GFP-ENA1*-overexpressed rice plants and WT.

Figure S3 Insertion positions and *ENA1* expression in the mutants. **(A)** Schematic representation of *ENA1* and insertion positions of *tos17* fragments. Primer positions used for *tos17* insertion checks are represented by arrows. Blue arrows indicate primer Tos17L and black arrows indicated primers pE1, pE2, pE3, pE4, and pE5 (Supplementary Table S1). **(B,C)** Confirmation of integration of *tos17* **(B)** and the homozygous status of *ena1* mutants. **(B)** Nested PCR with primers; Tos17L primer located in *tos17* and pE1–5 located in the *ENA1* gene were used. **(C)** Primers pE1–5 located in *ENA1* interposing *tos17* were used. Fragments of 1.3, 3, or 1.6 kb were predicted to be amplified in NG1060, NC0379, and other lines, respectively, when *tos17* was not inserted. **(D)** RT-PCR was performed to verify the expression of *ENA1* in *ENA1 tos17* insertion lines.

Figure S4 Plant phenotypes of *ena1* knockout mutants (0379 #A and 1060 #A), *GFP-ENA1*- or *ENA1-GFP*-overexpressing rice plants, and WT. Rice plants were grown hydroponically under Fe-sufficient and Fe-deficient conditions. The Fe deficiency treatment lasted for 7 days. **(A)** Shoot length and root length. Error bars represent the standard error (*n* = 9). **(B)** Relative chlorophyll contents (SPAD values) of the newest yellow leaves and oldest green leaves. **(C,D)** Fe, copper (Cu), Zn, and Mn concentrations in the youngest yellow leaves **(C)** and oldest green leaves **(D)** of *ena1* knockout mutants, *GFP-ENA1*- or *ENA1-GFP*-overexpressing rice plants, and WT after 7 days of Fe-deficient treatment. Values represent means of three replicates. Error bars represent standard deviation. DW, dry weight. **(E)** Root appearance after 7 days of Fe deficiency. Scale bars represent 5 cm.

Figure S5 Gene ontology (GO) terms enriched in the list of genes, whose expression levels were downregulated in the roots of *ena1* knockout mutants compared to WT under Fe-deficient conditions. **(A)** Biological process. **(B)** Molecular function. **(C)** Cellular component.

Figure S6 GO terms enriched in the list of genes, whose expression levels were downregulated in the shoots of *ena1* knockout mutants compared to WT under Fe-deficient conditions. **(A)** Biological process. **(B)** Molecular function. **(C)** Cellular component.

Figure S7 GO terms enriched in the list of genes, whose expression levels were downregulated in the roots of *ena1* knockout mutants compared to WT under Fe-sufficient conditions. **(A)** Cellular component.

Figure S8 GO terms enriched in the list of genes, whose expression was downregulated in the shoots of *ena1* knockout mutants compared to WT under Fe-sufficient conditions. **(A)** Molecular function. **(B)** Cellular component.

Table S1 Primers used in the analysis of *ENA1* tos17 insertion lines.

Table S2 Oligo DNA microarray analysis of ena1 knockout mutant lines.
