# Supplementary figures and images for: Characterization of the Nicotianamine Exporter ENA1 in Rice
Source: Front Plant Sci. 2019 Apr 30;10:502. doi: 10.3389/fpls.2019.00502 (PMC6503003; doi:10.3389/fpls.2019.00502)

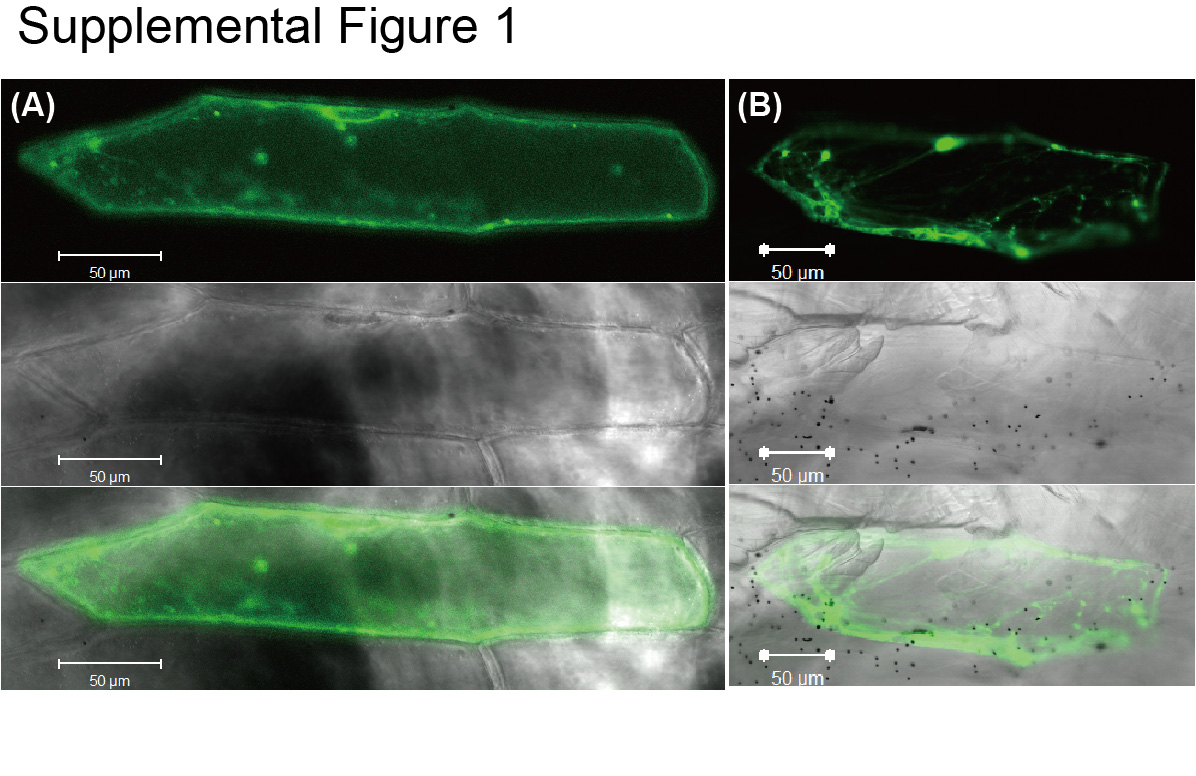

Supplement: Figure S1 — Subcellular localization of ENA1 in onion epidermal cells. (A) GFP-ENA1. (B) ENA1-GFP. [file Image_1.JPEG]

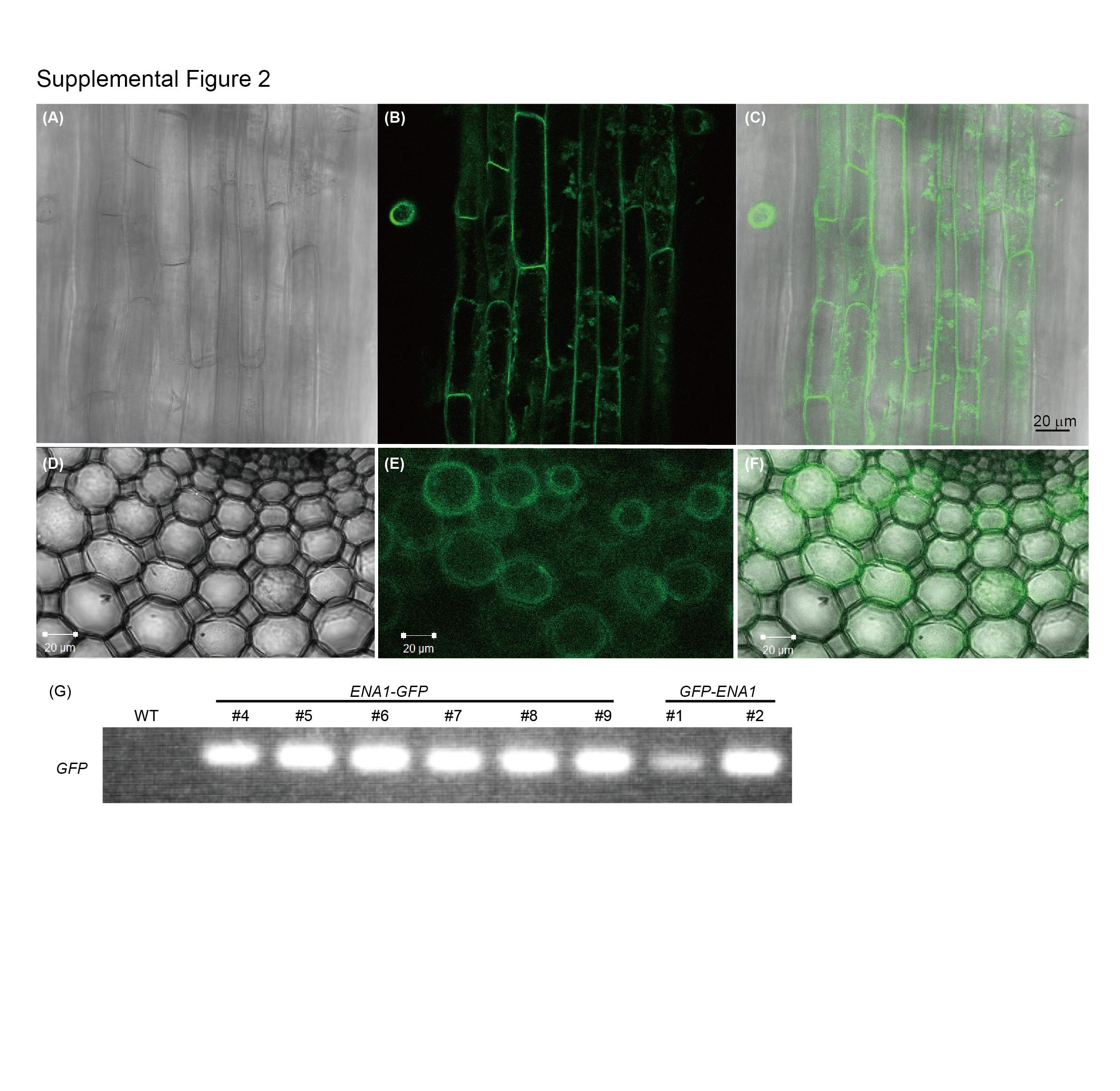

Supplement: Figure S2 — Subcellular localization of ENA1-GFP in rice roots. (A,D) Differential interference contrast image. (B,E) Fluorescence image. (C,F) Overlay. (G) GFP expression was examined by RT-PCR in ENA1-GFP- or GFP-ENA1-overexpressed rice plants and WT. [file Image_2.JPEG]

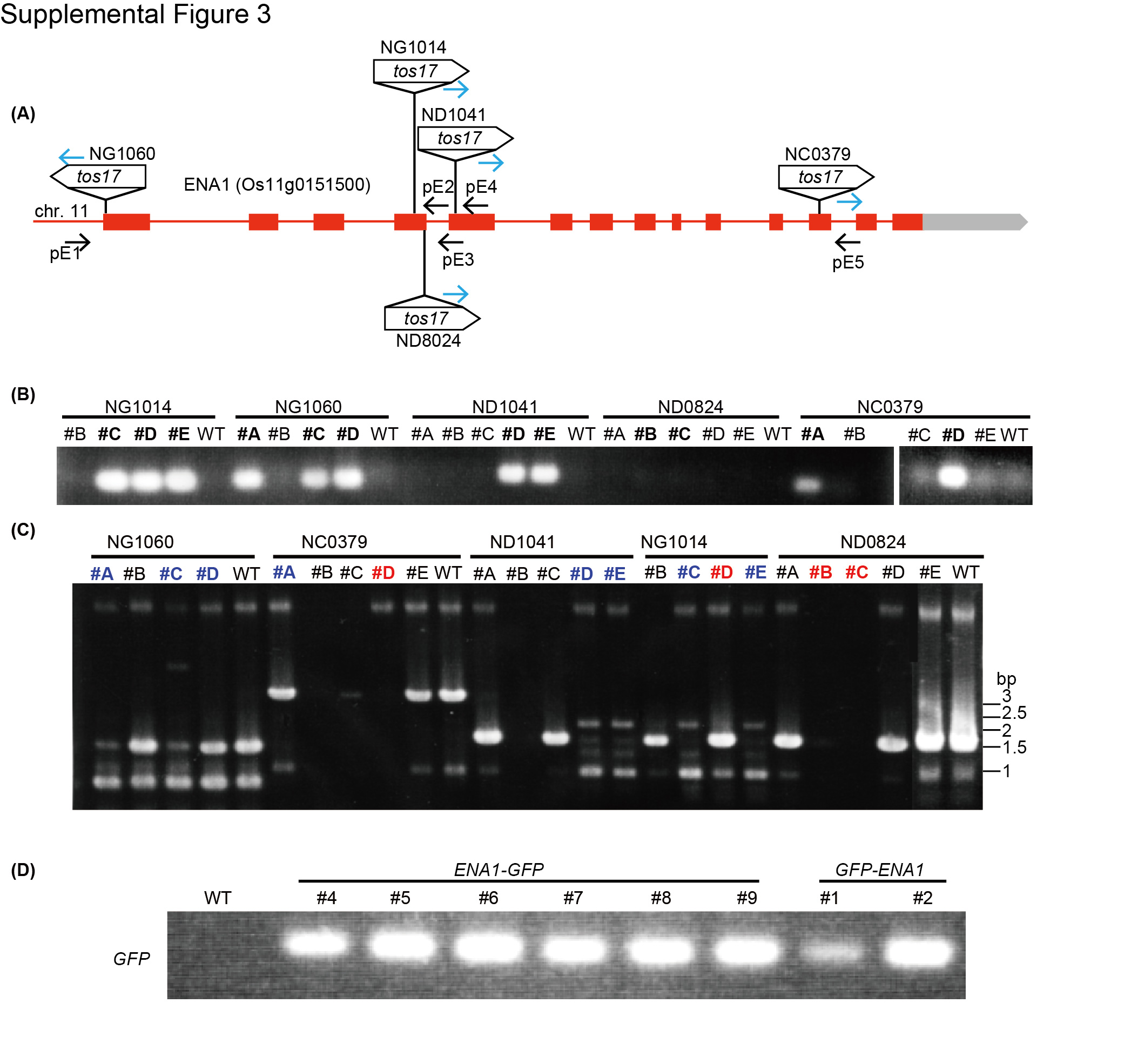

Supplement: Figure S3 — Insertion positions and ENA1 expression in the mutants. (A) Schematic representation of ENA1 and insertion positions of tos17 fragments. Primer positions used for tos17 insertion checks are represented by arrows. Blue arrows indicate primer Tos17L and black arrows indicated primers pE1, pE2, pE3, pE4, and pE5 (Supplementary Table S1). (B,C) Confirmation of integration of tos17 (B) and the homozygous status of ena1 mutants. (B) Nested PCR with primers; Tos17L primer located in tos17 and pE1–5 located in the ENA1 gene were used. (C) Primers pE1–5 located in ENA1 interposing tos17 were used. Fragments of 1.3, 3, or 1.6 kb were predicted to be amplified in NG1060, NC0379, and other lines, respectively, when tos17 was not inserted. (D) RT-PCR was performed to verify the expression of ENA1 in ENA1 tos17 insertion lines. [file Image_3.JPEG]

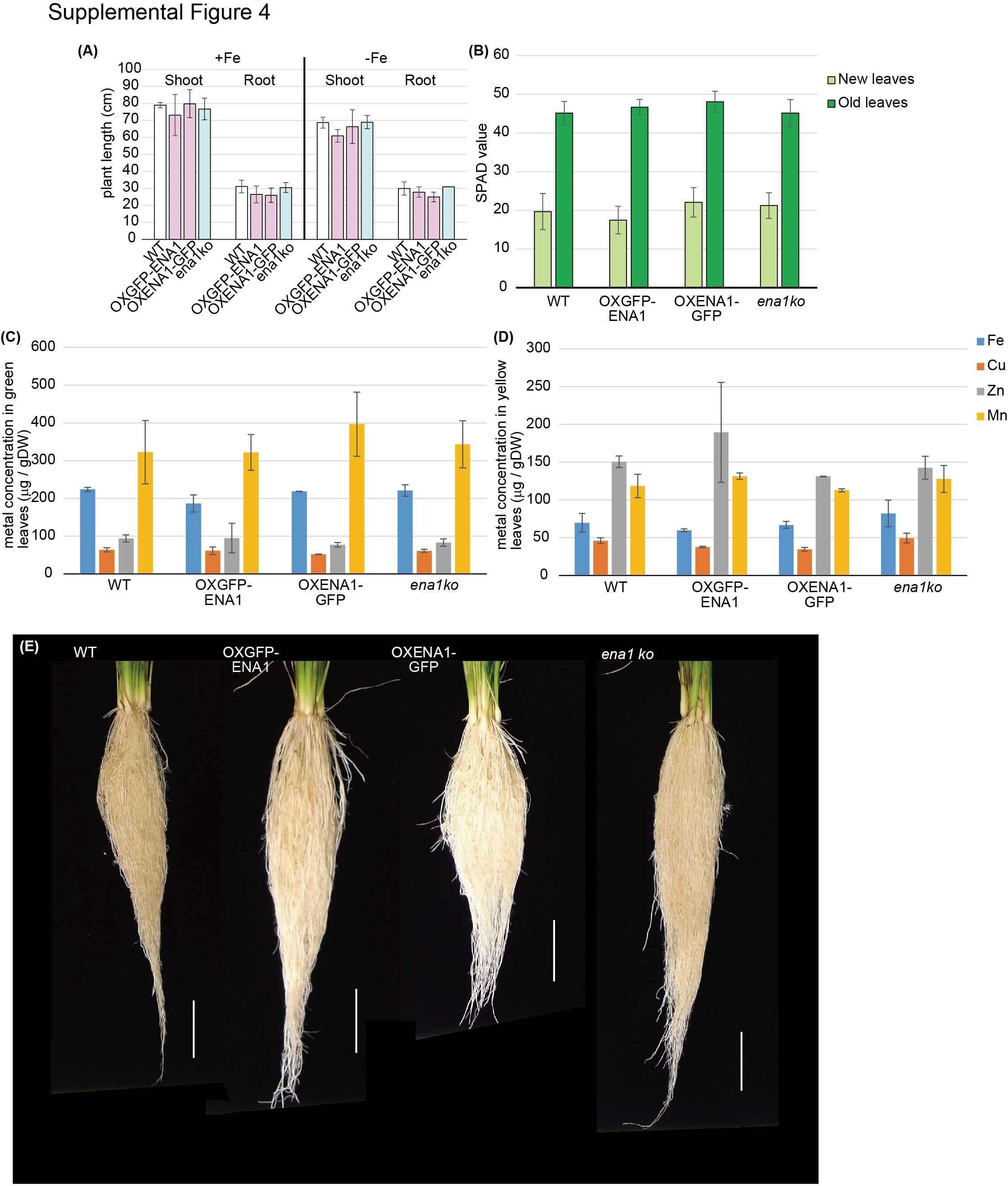

Supplement: Figure S4 — Plant phenotypes of ena1 knockout mutants (0379 #A and 1060 #A), GFP-ENA1- or ENA1-GFP-overexpressing rice plants, and WT. Rice plants were grown hydroponically under Fe-sufficient and Fe-deficient conditions. The Fe deficiency treatment lasted for 7 days. (A) Shoot length and root length. Error bars represent the standard error (n = 9). (B) Relative chlorophyll contents (SPAD values) of the newest yellow leaves and oldest green leaves. (C,D) Fe, copper (Cu), Zn, and Mn concentrations in the youngest yellow leaves (C) and oldest green leaves (D) of ena1 knockout mutants, GFP-ENA1- or ENA1-GFP-overexpressing rice plants, and WT after 7 days of Fe-deficient treatment. Values represent means of three replicates. Error bars represent standard deviation. DW, dry weight. (E) Root appearance after 7 days of Fe deficiency. Scale bars represent 5 cm. [file Image_4.JPEG]

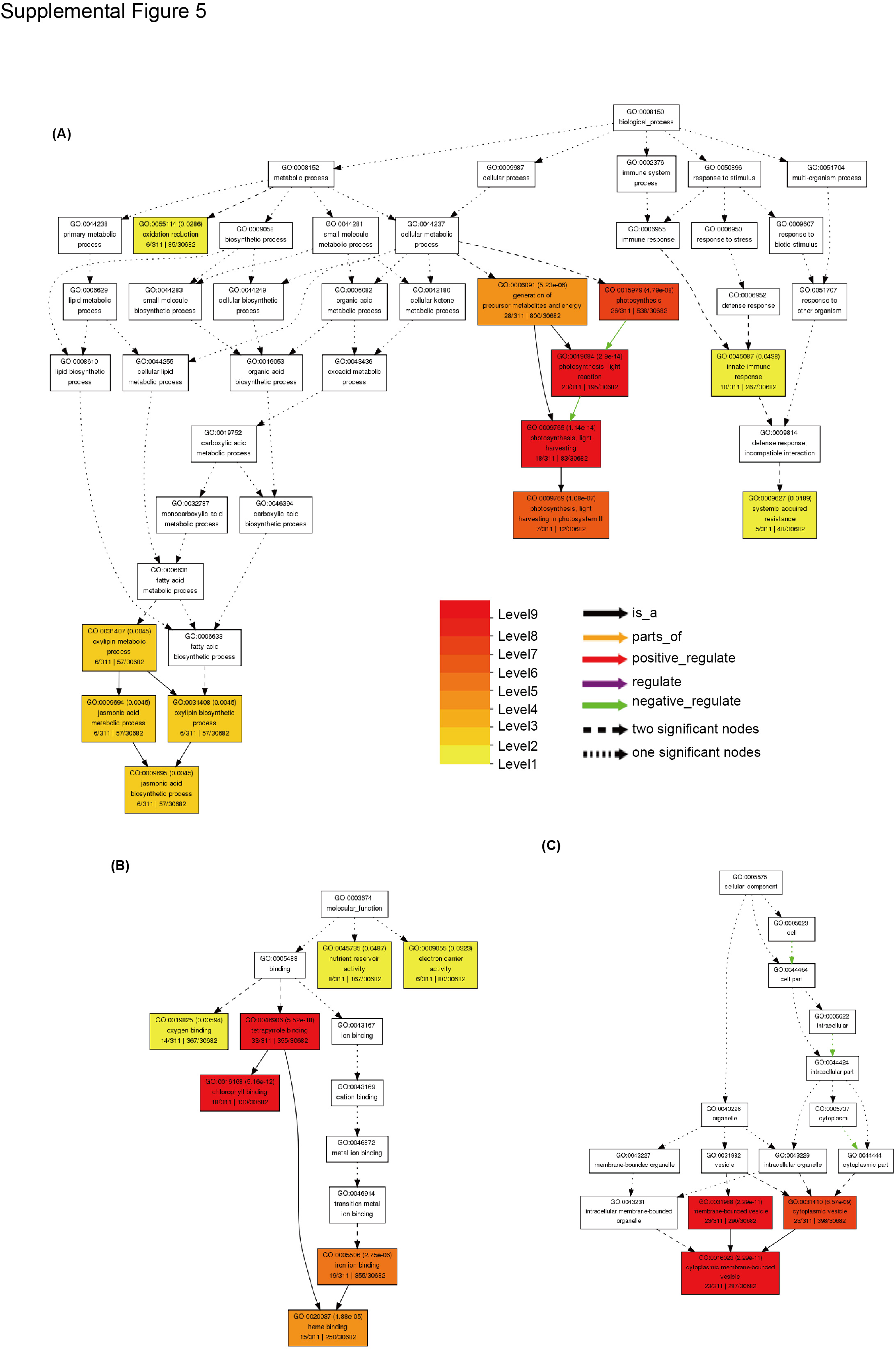

Supplement: Figure S5 — Gene ontology (GO) terms enriched in the list of genes, whose expression levels were downregulated in the roots of ena1 knockout mutants compared to WT under Fe-deficient conditions. (A) Biological process. (B) Molecular function. (C) Cellular component. [file Image_5.JPEG]

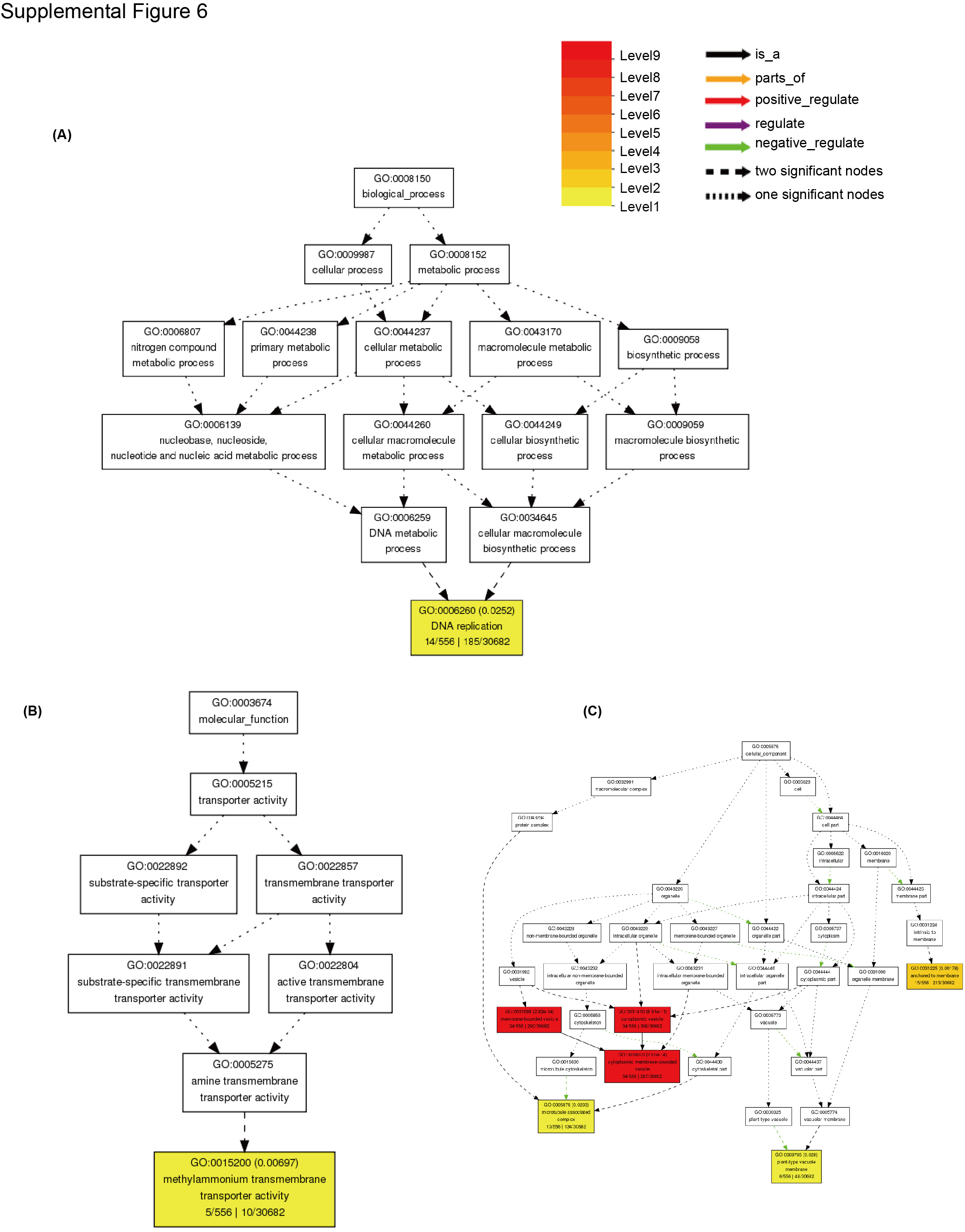

Supplement: Figure S6 — GO terms enriched in the list of genes, whose expression levels were downregulated in the shoots of ena1 knockout mutants compared to WT under Fe-deficient conditions. (A) Biological process. (B) Molecular function. (C) Cellular component. [file Image_6.JPEG]

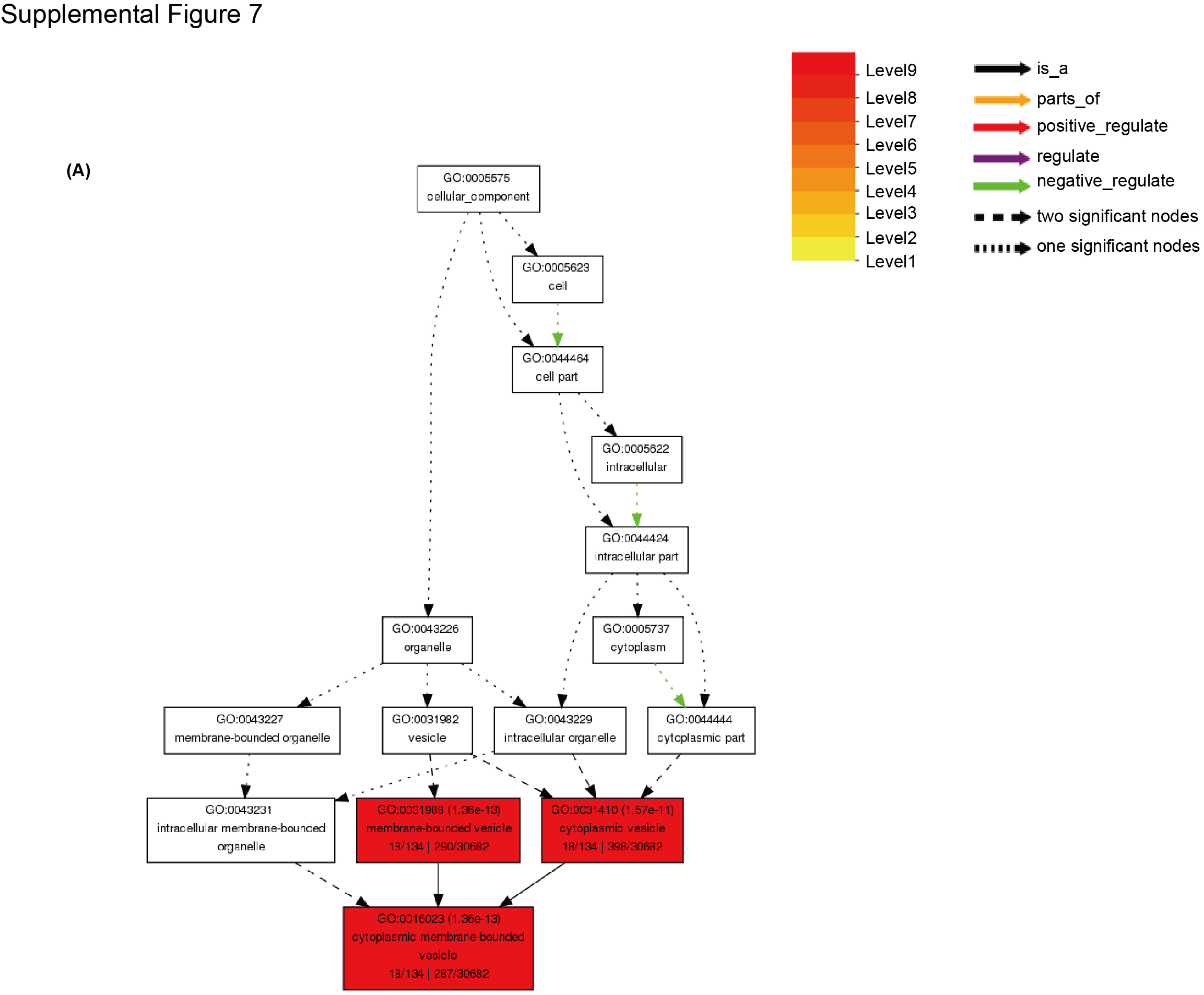

Supplement: Figure S7 — GO terms enriched in the list of genes, whose expression levels were downregulated in the roots of ena1 knockout mutants compared to WT under Fe-sufficient conditions. (A) Cellular component. [file Image_7.JPEG]

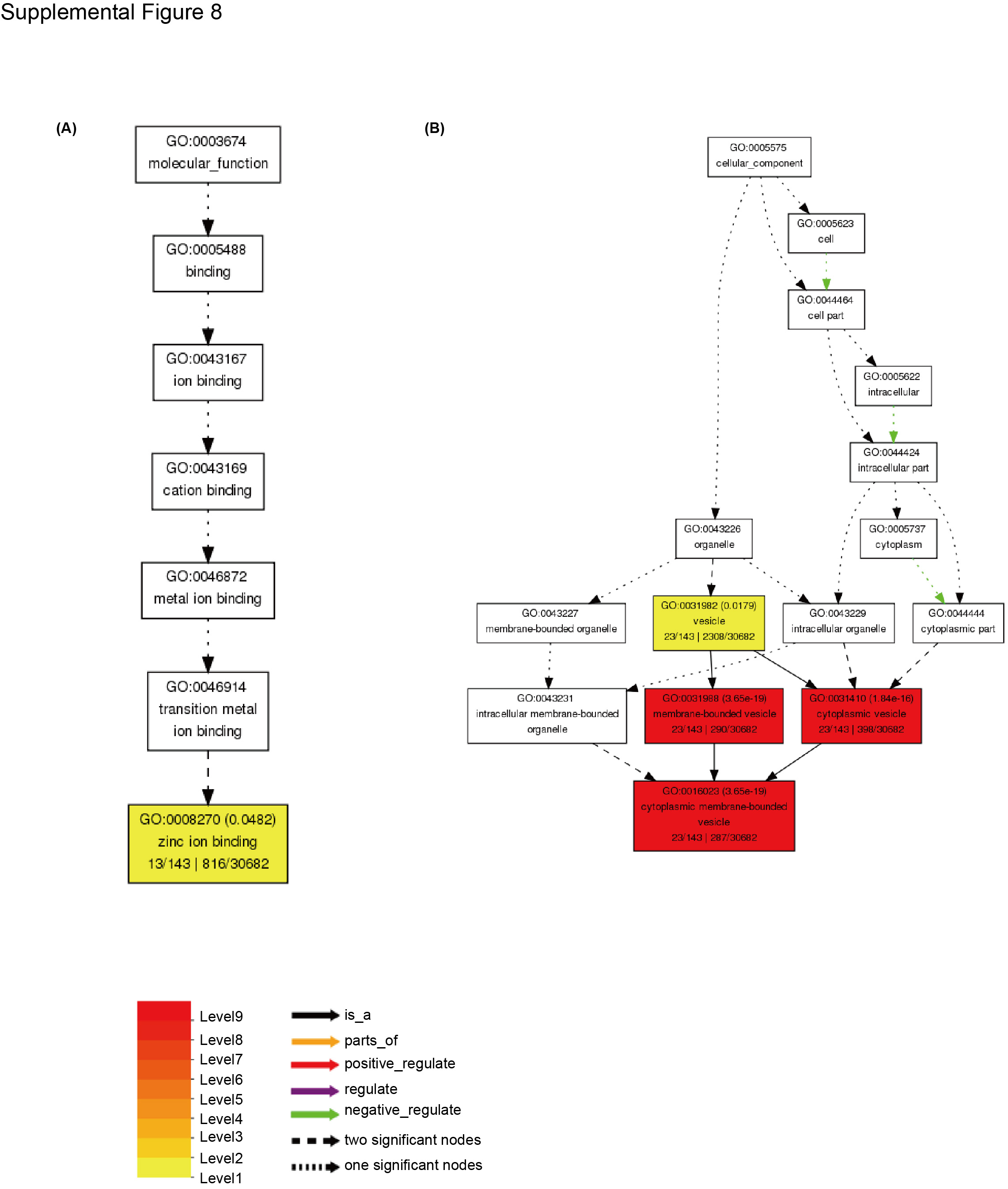

Supplement: Figure S8 — GO terms enriched in the list of genes, whose expression was downregulated in the shoots of ena1 knockout mutants compared to WT under Fe-sufficient conditions. (A) Molecular function. (B) Cellular component. [file Image_8.JPEG]
