# Supplementary material for: Characterization of the Nicotianamine Exporter ENA1 in Rice
Source: Front Plant Sci. 2019 Apr 30;10:502. doi: 10.3389/fpls.2019.00502 (PMC6503003; doi:10.3389/fpls.2019.00502)
Supplement: Table S1 — Primers used in the analysis of ENA1 tos17 insertion lines. [file Table_1.pdf]

Table 1. Primers used in the analysis of ENA1 tos17 insertion lines.

| Primer name | sequence |
|---|---|
| AK102457forTOS_FW | 5'-GATGCTAACTCTTGGTTTCA-3' |
| Tos17-L | 5' ATTGTTAGGTTGCAAGTTAGTTAAGA-3' |
| NC0379-R | 5' GACATGGCAATGCAACATTT-3' |
| ND1041-R | 5' TCCTTGATAGACTCCCAGCG-3' |
| ND8024-R | 5' AACCGAGACCTGATAATGCG-3' |
| NG1014-R | 5' CTAGAGAAGCCATTGCCCAG-3' |
| NG1060-R | 5' TTCTGAAGCCACTGATGCAC-3' |
